# Machine Learning Models to Predict Bone Metastasis Risk in Patients With Lung Cancer

**DOI:** 10.1002/cam4.70383

**Published:** 2024-11-18

**Authors:** Kevin Wang Leong So, Evan Mang Ching Leung, Tommy Ng, Rachel Tsui, Jason Pui Yin Cheung, Siu‐Wai Choi

**Affiliations:** ^1^ Department of Orthopaedics and Traumatology, School of Clinical Medicine, Li Ka Shing Faculty of Medicine The University of Hong Kong Hong Kong

**Keywords:** Bone metastasis, Lung cancer, Machine learning prediction

## Abstract

**Introduction:**

The aim of this study was to find the most appropriate variables to input into machine learning algorithms to identify those patients with primary lung malignancy with high risk for metastasis to the bone.

**Patient Inclusion:**

Patients with either histological or radiological diagnoses of lung cancer were included in this study.

**Results:**

The patient cohort comprised 1864 patients diagnosed from 2016 to 2021. A total of 25 variables were considered as potential risk factors. These variables have been identified in previous studies as independent risk factors for bone metastasis. Treatment methods for lung cancer were taken into account during model development. The outcome variable was binary, (presence or absence of bone metastasis) with follow‐up until death or 12‐month survival, whichever is the sooner. Results showed that American Joint Committee on Cancer staging, the use of EGFR inhibitor, age, T‐staging, and lymphovascular invasion were the five input features contributing the most to the model algorithm. High AJCC staging (OR 1.98; *p* < 0.05), the use of EGFR inhibitor (OR 6.14; *p* < 0.05), high T‐staging (OR 1.47; *p* < 0.05), and the presence of lymphovascular invasion (OR 4.92; *p* < 0.05) increase predicted risk of bone metastasis. Conversely, older age reduces predicted bone metastasis risk (OR 0.98; *p* < 0.05).

**Conclusion:**

The machine learning model developed in this study can be easily incorporated into the hospital's Clinical Management System so that input variables can be immediately utilized to give an accurate prediction of bone metastatic risk, therefore informing clinicians on the best treatment strategy for that individual patient.

**Abbreviations**
ADASYN: Adaptive Synthetic Sampling Approach
AJCC: American Joint Committee on Cancer
BM: bone metastasis
CMS: clinical management system
COV: coefficient of variation
FDG‐PET‐CT: fluorodeoxyglucose‐positron emission tomography‐CT
kNN: k‐nearest neighbor
LDA: linear discriminant analysis
ML: machine learning
MLP‐BP: multilayer perceptron with backpropagation
NCCN: National Comprehensive Cancer Network
NPV: negative predictive value
NSCLC: nonsmall cell lung cancer
RBF: radial basis function
SEER: Surveillance, Epidemiology and End Results
SHAP: Shapley additive explanations
SMOTE: Synthetic Minority Oversampling Technique
SP: specificity
SRE: skeletal‐related event
SVM: support vector machines
TRIPOD: Transparent Reporting of a Multivariable Prediction Model for Individual Prognosis or Diagnosis

## 1 Introduction

The most common cause of cancer‐related deaths globally is lung cancer. Global Cancer Observatory estimates in 2020 showed that Asia suffered the highest burden of lung cancer cases, with 60% of all new cases (1,315,136 cases) reported in the region [1]. Additionally, 62% of all deaths from lung cancer (1,112,517 cases) occurred in Asia [1]. At the point of diagnosis, approximately 20%–30% of nonsmall cell lung cancer patients present with metastatic disease [2]. In particular, approximately 30% to 40% of patients with advanced lung cancer are estimated to experience bone metastases [3]. Bone metastases can cause skeletal‐related events (SREs), including bone pain, pathological fractures, spinal cord compression, and hypercalcemia, which can have a major negative impact on the quality of life and survival of patients. Patients with bone metastases survive for a median of less than 6 months [3]. Therefore, timely detection and treatment of bone metastases is imperative in patients with lung cancer.

Currently, imaging techniques are used to detect bone metastasis, but these methods either have a limited sensitivity or may result in unnecessary radiation exposure [4, 5]. National Comprehensive Cancer Network (NCCN) screening guidelines have discouraged clinicians from exposing asymptomatic patients to imaging assessments [6]. Furthermore, bisphosphonates, such as zoledronic acid and ibandronate, have been shown to be effective in reducing skeletal‐related events (SREs) and pain associated with bone metastases in lung cancer patients, with only few adverse effects [7]. Yet, bone metastases currently adversely affects so many patients with lung malignancy. Therefore, there is an urgent need for an effective and easily accessible tool for early diagnosis and treatment of lung cancer bone metastases.

Nomogram to predict bone metastases in patients with different histological types of lung cancer based on logistic regression analysis have been developed [8, 9], and although these tools are useful in countries with limited resources, this method is inheritably limited by its inability to analyze large and diverse datasets common in the clinical arena [10]. Compared with traditional logistical modeling, machine learning (ML) can reveal valuable patterns and associations that may be challenging for even highly skilled clinical individuals to identify in large datasets [10]. In recent years, two prediction models using machine learning for lung cancer bone metastases have been developed [9, 11]. Both of these models only incorporated data from the Surveillance, Epidemiology and End Results (SEER) database, therefore important parameters relevant to lung cancer including smoking status were not included as the models are limited by the data collected by SEER. The objective of this current study, therefore, was to develop a reliable clinical model with high precision, incorporating all relevant variables routinely collected by the various specialties which are involved in the treatment of patients with lung malignancies.

## 2 Materials and Methods

### 2.1 Data Description

All electronic health records were extracted from the clinical management system (CMS) under the Hospital Authority (HA) of Hong Kong. Patients with either histological or radiological diagnoses of lung cancer were retrospectively included in this study. Patients with secondary lung cancers or presented with bone metastasis in the initial diagnosis were excluded. Ultimately, the patient cohort comprised 1864 patients from a territory‐wide, tertiary referral center, diagnosed from 2016 to 2021 (census date at 2021 to allow for follow‐up information).

### 2.2 Variable Selection and Rationale

A total of 25 variables, spanning sociodemographic, clinical, pathological, and treatment information, were considered as potential risk factors of bone metastasis. These variables have been identified in previous studies as independent risk factors for bone metastasis [8, 9, 10, 11]. The treatment methods for lung cancer, including surgery, chemotherapy, radiotherapy, targeted therapy, and immunotherapy, were taken into account during model development. The outcome variable was binary, indicating the presence or absence of bone metastasis in all patients with a minimum follow‐up of 12 months or till death, whichever is the soonest. This study did not consider specific statistical assessments of how these variables affect the outcome within this cohort.

## 3 Model Development

### 3.1 Data Preprocessing and Feature Engineering

Electronic spreadsheet templates in Microsoft Excel were used for data collection, followed by column filtering to retain relevant variables showing correlations. Input features for training included continuous [1], Boolean [12], and categorical [6] variables. In total, 25 variables were used to build a machine learning model to predict bone metastasis risk. The variables used in the machine learning models are marked with an asterisk on Table 1. Arbitrary value imputation was employed to manage missing values [13].

**TABLE 1:** Clinical and pathological characteristics of patients with or without bone metastasis.

Variables	Without bone metastasis	With bone metastasis	*p*
	*N* = 1537	*N* = 327	
	*N* (%)	*N* (%)	
Mean age at diagnosis (years)^a^	65.4	67.6	0.15
Mean duration of follow‐up (years)^a^	5.04	5.73	0.18
Sex^a^			0.11
Male	846 (55%)	196 (59.9%)	
Female	691 (45%)	131 (40.1%)	
Lung cancer type^a^			< 0.01
Adenocarcinoma	1231 (80%)	241 (73.7%)	
Squamous cell carcinoma	157 (10.2%)	23 (7.0%)	
Adenosquamous cell carcinoma	1 (0.07%)	0	
Mucoepidermoid carcinoma	1 (0.07%)	0	
Sarcomatoid carcinoma	1 (0.07%)	0	
Nonsmall cell carcinoma, unclassified	58 (3.77%)	34 (10.4%)	
Small cell carcinoma	32 (2.08%)	11 (3.36%)	
Unknown	55 (3.58%)	18 (5.50%)	
Smoking status^a^			0.30
Nonsmoker	819 (53.2%)	157 (48%)	
Smoker	318 (20.7%)	81 (24.8%)	
Ex‐smoker	382 (24.9%)	85 (26.0%)	
Unknown	18 (1.17%)	4 (1.22%)	
Comorbidities			
Hypertension^a^	728 (47.4%)	141 (43.1%)	0.33
Diabetes mellitus^a^	319 (20.8%)	54 (16.5%)	0.08
Chronic renal failure^a^	31 (2.02%)	4 (1.22%)	0.34
Nonsurgical treatment for primary tumor			
Chemotherapy^a^	316 (20.6%)	141 (43.1%)	< 0.01
Radiotherapy^a^	271 (17.6%)	113 (34.6%)	< 0.01
Anti‐EGFR^a^	144 (9.37%)	127 (38.8%)	< 0.01
ALK inhibitor^a^	13 (0.846%)	3 (0.917%)	0.90
PD1/PDL1 inhibitor^a^	45 (2.92%)	24 (7.34%)	< 0.01
Anti‐VEGF^a^	3 (0.195%)	5 (1.53%)	< 0.01
CTLA inhibitor^a^	1 (0.07%)	2 (0.611%)	0.03
Drug trial^a^	2 (0.13%)	3 (0.917%)	0.01
Surgery for primary tumor			
Lobectomy^a^	906 (58.9%)	79 (24.2%)	< 0.01
Wedge resection^a^	170 (11.1%)	22 (6.73%)	0.02
Segmentectomy^a^	16 (1.04%)	2 (0.611%)	0.47
Others^a^	17 (1.11%)	5 (1.53%)	0.52
No surgery^a^	592 (39.5%)	219 (67.0%)	< 0.01
AJCC Cancer staging^a^			< 0.01
I	705 (45.9%)	39 (11.9%)	
II	150 (9.76%)	18 (5.50%)	
III	167 (10.87%)	49 (15.0%)	
IV	135 (8.78%)	163 (49.8%)	
Unknown	380 (24.7%)	58 (17.7%)	
T‐staging^a^			< 0.01
T in situ	85 (5.53%)	0	
T1	662 (43.0%)	38 (11.6%)	
T2	238 (15.5%)	41 (12.5%)	
T3	112 (7.29%)	17 (5.20%)	
T4	63 (4.10%)	28 (8.56%)	
Unknown	377 (24.5%)	20 (6.12%)	
Lymphovascular invasion^a^			< 0.01
Yes	222 (14.4%)	59 (18.0%)	
No	797 (51.9%)	43 (13.1%)	
Unknown	518 (33.7%)	225 (68.8%)	
Differentiation grade^a^			< 0.01
Well	101 (6.57%)	7 (2.14%)	
Moderate	652 (42.4%)	57 (17.4%)	
Poor	170 (11.0%)	39 (11.9%)	
Unknown	614 (39.9%)	224 (68.5%)	
Resection margin^a^			< 0.01
Clear	910 (59.2%)	82 (25.1%)	
Unclear	41 (2.67%)	12 (3.67%)	
Unknown	586 (38.1%)	233 (71.3%)	

^a^ Variables used in machine learning models.

### 3.2 Machine Learning Algorithms Considered

The performance of nine most commonly used machine learning classifiers were compared with select the most suitable models for prediction. These included logistic regression, linear support vector machines (SVM), radial basis function (RBF) kernel SVM, random forest, decision tree, gradient boosting, k‐nearest neighbor (kNN), linear discriminant analysis, and multilayer perceptron with backpropagation (MLP‐BP).

### 3.3 Model Training

Data were divided into training and test sets following the 80:20 rule. Tenfold crossvalidation was used to tune hyperparameters and evaluate model stability. Model stability, reflecting the sensitivity of machine learning models to variations in training data, was assessed using the coefficient of variation (COV). Lower COV values indicated better model stability.

Due to the low incidence of bone metastasis events in the dataset, the study compared two synthetic oversampling techniques, SMOTE and ADASYN, and class weight assignment for specific algorithms (logistic regression, SVM, decision tree, and random forest) to handle class imbalance. SMOTE and ADASYN created synthetic data for the minority class using the k‐nearest neighbor algorithm, with ADASYN adapting to density distributions. Class weight assignment automatically adjusted cost function weights based on class frequencies. In total, 23 different models were trained in this study.

Hyperparameter tuning was conducted manually for decision tree, random forest, kNN, and MLP‐BP and automatically for others based on crossvalidation performance.

### 3.4 Model Validation

The performance of trained models was assessed using an unseen 20% of the dataset for testing. Various performance metrics, including AUC value, accuracy, sensitivity, specificity, precision, negative predictive value, and F1‐score, were computed to compare overall performance and select the best model. F1 score took precedence as the primary metrics for evaluating model performance given the imbalanced dataset. However, in cases where F1‐scores were comparable (within a range of ± 0.05), a stronger focus was placed on maximizing sensitivity. This emphasis stemmed from the time‐sensitive nature of bone metastasis development, where reliable prediction models should effectively identify most cases with bone metastasis to facilitate timely management and more frequent follow‐up.

### 3.5 Explainability and Net Benefit Analyses

The Shapley additive explanations (SHAP) framework was utilized to interpret the rationale behind risk predictions made by the best‐performing models. This model‐agnostic approach provides insights into how input features contribute to predictions.

### 3.6 Computation

The study utilized SPSS for descriptive statistics, while Python with scikit‐learn was employed for training, testing, and validation of supervised learning algorithms, as well as for model deployment. Adherence to the Transparent Reporting of a Multivariable Prediction Model for Individual Prognosis or Diagnosis (TRIPOD) checklist was maintained throughout the study for transparent reporting.

## 4 Results

### 4.1 Patient Demographics

1864 patients were included in this study, of which 1537 (82.0%) patients did not develop bone metastasis (NBM) and 327 (18.0%) developed bone metastasis (BM) at the time of study census. The mean age of both groups of patients were similar (65.4 years old in NMB; 67.6 years old in BM). The majority of them were males (55.0% in NBM; 59.9% in BM) and around half were nonsmokers (53.2% in NMB; 48.0% in BM). The most common histological cancer type was adenocarcinoma (80.0% in NMB; 73.7% in BM). The majority of patients with NMB received surgery for the primary lung tumor (60.5%), compared with 33.0% patients with BM. The majority of patients in the NMB group were diagnosed with a Stage I lung cancer (45.9%), while the majority in the BM group were diagnosed with Stage IV lung cancer (49.8%). Detailed clinical characteristics of patients are shown in Table 1.

### 4.2 Comparison of Model Testing Performance

**GRAPH 1:**
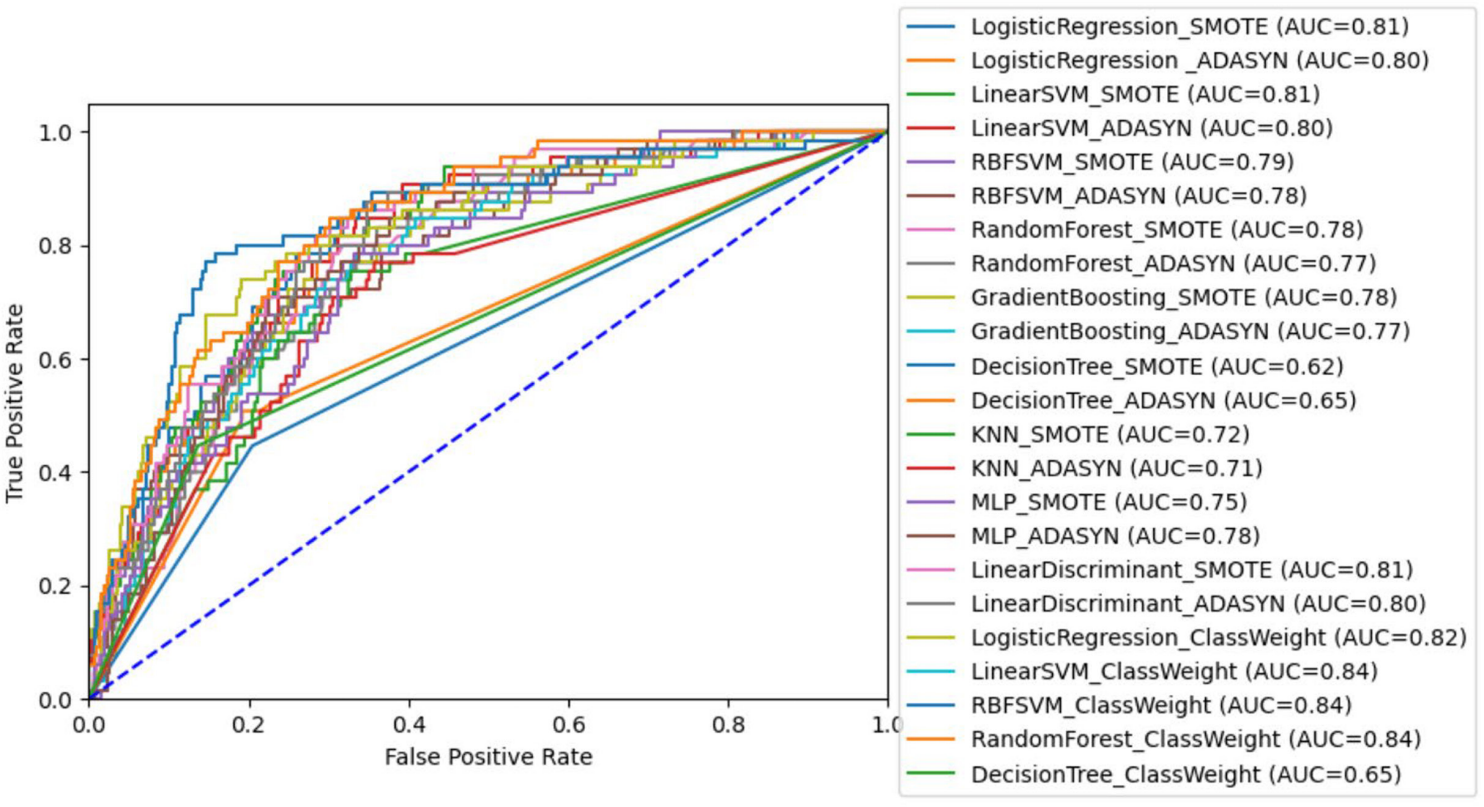
ROC curves comparing 23 training models.

Testing performance of all trained models are presented in Tables 2, 3, 4 and Graph 1. The performance of different models were heterogenous. Briefly, using logistic regression, the model based on class weight distribution achieved the highest accuracy of 0.85. With regard to linear SVM, model based on class weight distribution and SMOTE resulted in the same accuracy of 0.77. Furthermore, the model based on class weight distribution demonstrated a better F1 score and COA of 0.54 and 0.04, respectively. For RBF‐Kernel SVM, the model based on class weight distribution had a better accuracy and F1 score of 0.80 and 0.57, respectively. However, model based on SMOTE had the best stability with a COA of 1.84. Lastly, linear SVM, RBF‐Kernel SVM and random forest based on class weight distribution had the best AUC value of 0.84.

**TABLE 2:** Performance of nine machine learning classifiers on training and testing datasets using SMOTE and ADASYN.

Algorithms	SMOTE (Synthetic Minority Oversampling Technique)								
	Training			Testing					
	Mean accuracy	Standard deviation	Range	Accuracy	Sensitivity	Precision	F1 score	SP	NPV
Logistic regression	0.77	0.010	0.040	0.76	0.66	0.38	0.49	0.78	0.92
Linear SVM	0.77	0.012	0.033	0.77	0.68	0.40	0.50	0.79	0.92
RBF‐Kernel SVM	0.85	0.016	0.054	0.78	0.60	0.41	0.48	0.81	0.91
Random forest	0.88	0.023	0.073	0.78	0.51	0.40	0.45	0.84	0.89
Gradient boosting	0.88	0.30	0.11	0.79	0.43	0.39	0.41	0.86	0.88
Decision tree	0.81	0.020	0.068	0.73	0.51	0.32	0.40	0.78	0.88
kNN	0.84	0.023	0.085	0.73	0.63	0.35	0.45	0.75	0.94
MLP‐BP	0.85	0.021	0.062	0.76	0.46	0.35	0.40	0.82	0.88
LDA	0.77	0.011	0.037	0.76	0.71	0.40	0.51	0.78	0.93
	ADASYN								
Logistic regression	0.74	0.021	0.059	0.73	0.74	0.36	0.48	0.72	0.93
Linear SVM	0.73	0.023	0.075	0.75	0.68	0.38	0.49	0.77	0.92
RBF‐Kernel SVM	0.84	0.021	0.060	0.77	0.57	0.39	0.46	0.81	0.90
Random forest	0.88	0.020	0.060	0.78	0.54	0.40	0.46	0.83	0.90
Gradient boosting	0.88	0.015	0.056	0.77	0.45	0.37	0.41	0.84	0.88
Decision tree	0.81	0.024	0.068	0.74	0.51	0.34	0.40	0.79	0.88
kNN	0.83	0.020	0.065	0.71	0.62	0.33	0.43	0.73	0.90
MLP‐BP	0.85	0.0090	0.032	0.77	0.34	0.34	0.34	0.86	0.86
LDA	0.74	0.020	0.63	0.74	0.71	0.34	0.48	0.74	0.92

Abbreviations: ADASYN, Adaptive Synthetic Sampling Approach; kNN, k‐nearest neighbor; LDA, linear discriminant analysis; MLP‐BP, multilayer perceptron with backpropagation; NPV, negative predictive value; RBF radial basis function; SMOTE, Synthetic Minority Oversampling Technique; SP, specificity; SVM support vector machines.

**TABLE 3:** Performance of five machine learning classifiers on training and testing datasets using class weights.

Algorithms	Training			Testing					
	Mean accuracy	Standard deviation	Range	Accuracy	Sensitivity	Precision	F1 score	SP	NPV
Logistic regression	0.83	0.020	0.074	0.85^b^	0.31	0.63	0.41	0.96	0.87
Linear SVM	0.76	0.030	0.11	0.77	0.77	0.40	0.53	0.76	0.94
RBF‐Kernel SVM^a^	0.51	0.055	0.20	0.80	0.78^b^	0.45	0.57^b^	0.80	0.95^b^
Random forest	0.82	0.020	0.067	0.83	0.57	0.52	0.54^b^	0.89^b^	0.86
Decision tree	0.78	0.040	0.11	0.79	0.42	0.40	0.41	0.87	0.88

Abbreviations: kNN, k‐nearest neighbor; LDA, linear discriminant analysis; MLP‐BP, multilayer perceptron with backpropagation; NPV, negative predictive value; RBF, radial basis function; SP, specificity; SVM, support vector machines.

^a^ Algorithm with the best overall performance.

^b^ Best performance in the corresponding outcomes among algorithms.

**TABLE 4:** Stability of nine machine learning classifiers on training and testing datasets using SMOTE, ADASYN, and class weights.

Models	Coefficient of variation (COV)		
	SMOTE (Synthetic Minority Oversampling Technique)	ADASYN (Adaptive Synthetic Sampling Approach)	Class weight model
Logistic regression	1.42	2.83	2.36
Linear SVM	1.51	3.15	0.040
RBF‐Kernel SVM	1.84	2.57	1.07
Random forest	2.62	2.33	2.44
Decision tree	2.06	2.99	3.06
Gradient boosting	3.38	1.68	N/A
kNN	2.78	2.44	N/A
MLP‐BP	2.45	1.03	N/A
LDA	1.40	2.72	N/A

Abbreviations: ADASYN, Adaptive Synthetic Sampling Approach; kNN, k‐nearest neighbor; LDA, linear discriminant analysis; MLP‐BP, multilayer perceptron with backpropagation; RBF, radial basis function; SMOTE, Synthetic Minority Oversampling Technique; SVM, support vector machines.

Overall, models based on class weight distribution achieved better F1 scores, accuracies and AUC values than models based on either SMOTE or ADASYN. Regarding models based on class weight distribution, while logistic regression had the best accuracy of 0.85, RBF‐Kernel SVM had the best F1‐score of 0.57. When comparing RBF‐Kernel SVM and logistic regression, RBF‐Kernel SVM had a much better sensitivity (0.80) than logistic regression (0.57). Furthermore, due to the prioritization of F1 score over accuracy and sensitivity over precision, RBF‐Kernel SVM based class weight distribution would be considered to show the best performance for the prediction of bone metastasis in this study.

### 4.3 Explainability Analysis and Logistic Regression

**FIGURE 1:**
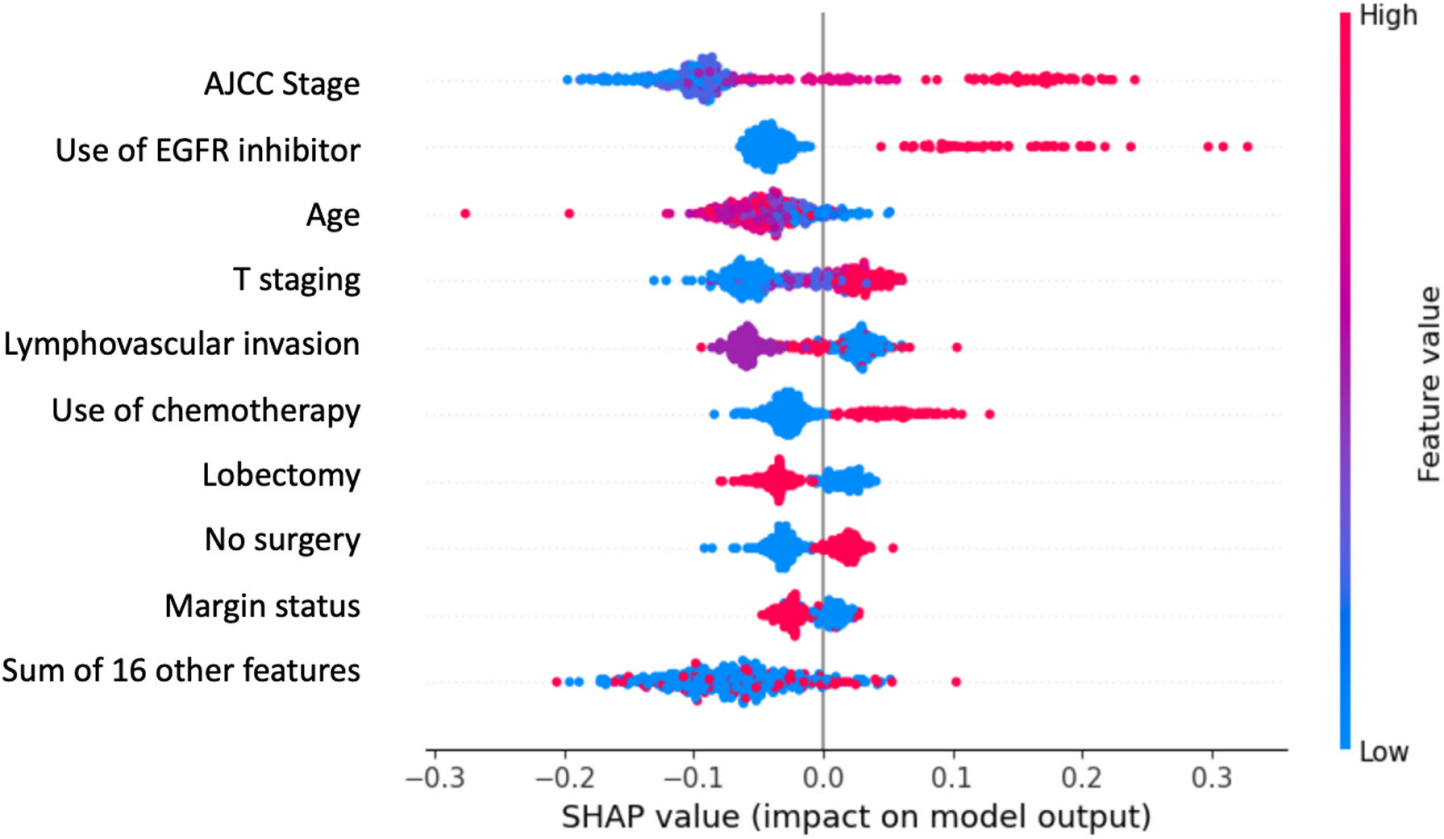
Global SHAP analysis for RBF‐Kernel SVM model. AJCC staging/T‐staging: Higher features value equals higher staging; Age: Higher features value equals higher age; Use of EGFR inhibitor/use of chemotherapy/lobectomy/no surgery: Red dot equals yes, blue dot equals no; Lymphovascular invasion: Red dot equals yes, purple dot equals no, blue dot equals no surgery performed; Margin status: Red dot equal unclear margin, purple dot equals clear margin, blue dot equals no surgery performed.

Explainability analysis using global SHAP values for RBF‐Kernel SVM is presented in Figure 1. American Joint Committee on Cancer (AJCC) staging, the use of EGFR inhibitor, age, T‐staging, and lymphovascular invasion were the five input features contributing the most to the model algorithm. Logistic regression for the top five input features is presented in Table 5. High AJCC staging (odds ratio 1.98; *p* < 0.001), the use of EGFR inhibitor (odds ratio 6.14; *p* < 0.001), high T‐staging (odds ratio 1.47; *p* < 0.001), and the presence of lymphovascular invasion (odds ratio 4.92; *p* < 0.001) increase predicted bone metastasis risk. Conversely, older age reduces bone metastasis risk (odds ratio 0.98; *p* < 0.001).

**TABLE 5:** Logistic regression model of the five most important input features.

Feature	Odds ratio [95% CI]	*p*
AJCC staging	1.98 [1.81, 2.17]	< 0.01
Use of EGFR inhibitor	6.14 [4.64, 8.14]	< 0.01
Age	0.980 [0.969, 0.911]	< 0.01
T‐staging	1.47 [1.37, 1.58]	< 0.01
Lymphovascular invasion^a^	4.92 [3.23, 7.50]	< 0.01

^a^ Patients with no data for lymphovascular invasion were not included.

## 5 Discussion

Lung cancer is the leading cause of cancer deaths worldwide [14]. In particular, up to 40% of patients with advanced lung cancer will develop bone metastasis, which significantly adversely impacts both survival and quality of life [3]. In this study, 17.5% of patients with lung cancer diagnosed from 2016 to 2021 developed bone metastases in the course of their disease, reflecting the high burden of bone involvement. It is therefore crucial to effectively screen patients who are at risk of developing bone metastases, which can guide overall treatment strategy.

Currently, clinical guidelines recommend fluorodeoxyglucose‐positron emission tomography‐CT (FDG‐PET‐CT) to detect distant metastases in patients with newly diagnosed lung cancer [15, 16]. However, the major limitations of FDG‐PET‐CT are its high cost, and high radiation exposure. It is therefore impractical to screen patients with lung cancers for distant metastases regularly and repeatedly. Thus, this study aimed to develop a robust prediction model for the early detection of lung cancer patients who are at a high risk of developing a bone metastasis.

The field of medicine is rapidly adopting machine learning due to the advancements in artificial intelligence technology. However, despite the significant progress made in utilizing various machine learning models, there is still ample room for improvement. Chen et al. developed a deep learning model to predict bone metastases in small cell lung cancer patients using data obtained from the publicly available SEER database [9]. Although the SEER database has many benefits for population‐based research, there are several limitations which have negatively impacted the accuracy of models based on this data [17]. Firstly, certain important variables including surgical margin, smoking status, the use of immunotherapy, and lymphovascular invasion are not available. Furthermore, the sequence of data collection cannot be verified from this database. Similarly, the machine learning model developed by Li et al. using the SEER database to predict bone metastasis in nonsmall cell lung cancer (NSCLC) is also subject to the same limitations [11]. Compared with their best model with a sensitivity of 0.74 and accuracy of 0.74, RBF‐Kernel SVM based on class weight distribution in our study had a better performance with a sensitivity and accuracy of 0.78 and 0.80 respectively. Teng et al. developed a random forest machine learning model using 11 serum indicators relating to the metabolism of bone metastasis to predict such in Stage IV NSCLC [18]. Although their random forest model achieved an accuracy of 0.94 and sensitivity of 0.92, the majority of input features in the study, including the serum indicators, were not measured routinely in standard clinical practice, limiting its translatability.

This study constructed 23 machine learning models using clinical data from Clinical Management System (CMS), a data collection system developed by Hong Kong Hospital Authority, and which incorporates all electronic clinical information and point of care documentation [12]. RBF‐Kernel SVM model based on class weights distribution showed the best performance in our study. Furthermore, our model using clinical variables achieved better sensitivity and accuracy when compared to other models developed using data from 50,581 NSCLC patients [11].

Global SHAP analysis showed that the most important input features in predicting bone metastasis were AJCC staging, the use of EGFR inhibitor, age, T‐staging, and lymphovascular invasion. TNM staging proposed by the AJCC is a widely used prognostic system in oncology. Although previous studies showed that using AJCC staging alone cannot achieve a high prediction accuracy of distant metastasis, our model demonstrated that the AJCC system ranked most importantly in terms of the SHAP value [19, 20]. It was also found that T‐staging was a strong predictor of bone metastases in this cohort, which is in line with previous investigations which found that although there was no correlation between tumor size and overall distant metastasis, patients with a tumor > 3 cm were at a higher risk of bone metastasis [21]. In addition, only the use of EGFR inhibitor, but not other systemic therapies, ranked in the top five most important variables in global SHAP analysis. Patients administered with EGFR inhibitors would be those with the EGFR mutation, which have been found to be closely associated with bone metastasis when compared to other molecular subgroups, or with the EGFR wild‐type group in multiple studies [22, 23, 24].

Interestingly, a younger age was also a strong predictor of bone metastasis in our study. We postulate that younger patients with lung malignancies generally have a better prognosis which increases the time to risk of developing bone metastasis. This postulation was supported by multiple population‐based studies on both lung and breast cancer [25, 26, 27]. Therefore, young patients with lung cancer warrant a more aggressive surveillance strategy.

Lastly, this current study showed that lymphovascular invasion was a predictive feature of bone metastasis. To date, multiple studies have demonstrated that the presence of lymphovascular invasion confer poor prognosis, including distant metastasis, in lung cancer [28, 29, 30, 31]. It was hypothesized that lymphovascular invasion is an early sign of micrometastasis which can result in subsequent vascular dissemination of tumor cells [32].

The results from this study can aid in the establishment of a scoring system which can inform clinicians about the risk of bone metastasis in the absence of a machine learning model. For example, patients with AJCC cancer Stage 4, the use of EGFR inhibitor, age above the mean of diagnosis (aged above 65), T‐staging 4, lymphovascular invasion, all these five factors will be given one point so that each patient would have a score out of five, five being the most at risk for bone metastasis, while patients with a score of zero would be at least risk.

There are two major limitations in our study. First, this is a retrospective study involving only patients from one hospital without external validation. Notably, more than 50% of patients in our cohort are never‐smokers, while only 20% of lung cancers patients worldwide are never‐smokers [33, 34]. Therefore, external validation is required to assess the reproducibility of our model. Second, the precision of our model is suboptimal. However, our model achieved a high sensitivity that is more important in risk stratification. Further studies to improve the precision is required, which may include the incorporation of other important features, including molecular profiles, biochemical markers, and imaging, into our model.

## 6 Conclusion

This study ascertains that machine learning models have satisfactory sensitivity and accuracy in identifying patients with lung cancer who are at risk of developing bone metastases using variables routinely collected at point of care in a major tertiary hospital. RBF‐Kernel SVM showed superior performance and has potential to serve as an important tool for the early diagnosis of bone metastases in lung cancer patients. Furthermore, the importance of input features using Global SHAP analysis was ranked which showed that demographic information, pathology of the primary lung malignancy, and treatment of the original tumor are all useful variables for predicting risk of bone metastases in lung cancer patients.

The machine learning model identified in this study can be easily incorporated into the hospital's Clinical Management System so that input variables can be immediately utilized to give an accurate prediction of bone metastatic risk, therefore informing clinicians on the best treatment strategy for that individual patient.

The strength of this study lies in that detailed, accurate and updated information was obtained from each individual patient. However, further external validation is required to assess the reproducibility of our model.

## Author Contributions

**Kevin Wang Leong So:** data curation (equal), formal analysis (equal), investigation (equal), writing – original draft (equal), writing – review and editing (equal). **Evan Mang Ching Leung:** data curation (equal), writing – original draft (equal), writing – review and editing (equal). **Tommy Ng:** data curation (equal), writing – original draft (supporting), writing – review and editing (supporting). **Rachel Tsui:** data curation (equal), writing – original draft (supporting), writing – review and editing (supporting). **Jason Pui Yin Cheung:** methodology (supporting), project administration (supporting), supervision (supporting), writing – original draft (supporting), writing – review and editing (supporting). **Siu‐Wai Choi:** conceptualization (lead), formal analysis (lead), project administration (lead), supervision (lead), validation (lead), writing – original draft (lead), writing – review and editing (lead).

## Ethics Statement

Approval to conduct this retrospective study was granted by the Institutional Review Board of the University of Hong Kong/Hospital Authority Hong Kong West Cluster (ref.: HKWC‐2022‐2921). All clinical data were anonymized by the researchers, and all potential patient identifiers were removed prior to data analysis.

Since this study is retrospective in nature and patients cannot be identified, the Institutional Review Board of the University of Hong Kong/Hospital Authority Hong Kong West Cluster waived the need for this study to obtain written or verbal consent from the patients.

## Conflicts of Interest

The authors declare no conflicts of interest.

## Data Availability

Data will be made available upon enquiry to the corresponding author.
